# Supplementary material for: Vitamin D Modified DSS-Induced Colitis in Mice via STING Signaling Pathway
Source: Biology (Basel). 2025 Jun 18;14(6):715. doi: 10.3390/biology14060715 (PMC12190092; doi:10.3390/biology14060715)
Supplement: Supplementary file 1 [file biology-14-00715-s001.zip › Supplementary Materials S1: 16s-report of gut microbiota/Groups/Lefse_Analysis/biomarkers_raw_images/1_d__Bacteria-p__Bacteroidota-c__Bacteroidia-o__Bacteroidales-f__Prevotellaceae-g__Prevotellaceae_NK3B31_group.pdf]

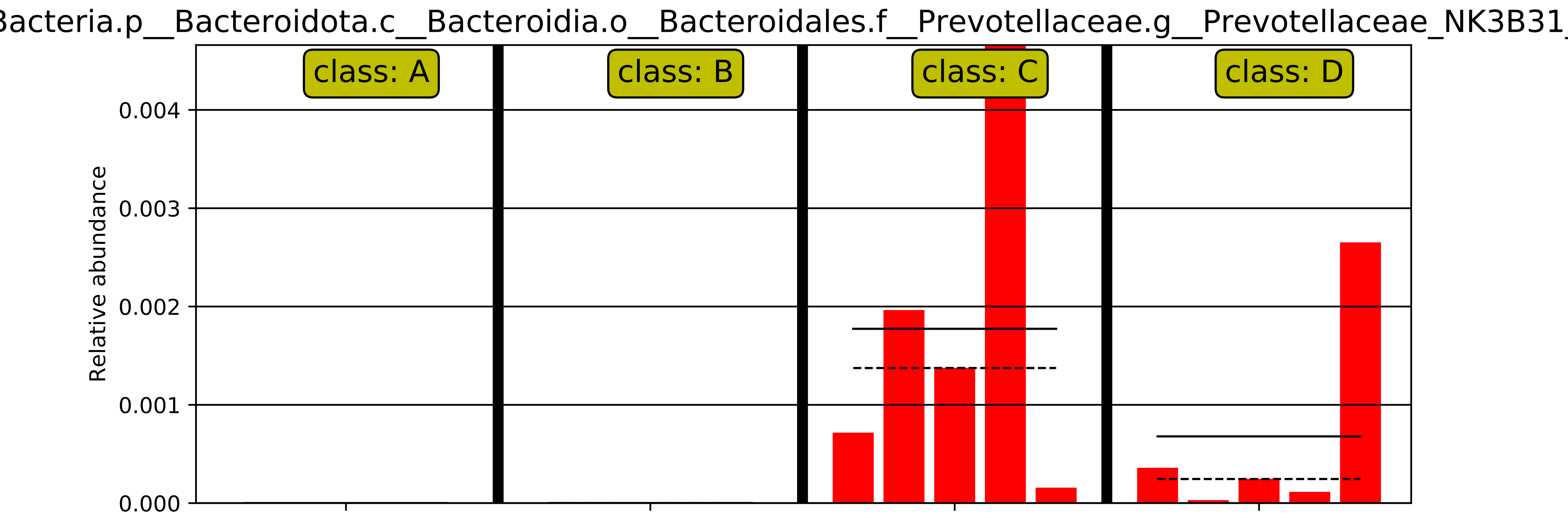

Bacteria.p__Bacteroidota.c__Bacteroidia.o__Bacteroidales.f__Prevotellaceae.g__Prevotellaceae_NK3B31
class: A
class: B
class: C
class: D
Relative abundance
0.000
0.001
0.002
0.003
0.004
